# Antibody-Hapten Recognition at the Surface of Functionalized Liposomes Studied by SPR: Steric Hindrance of Pegylated Phospholipids in Stealth Liposomes Prepared for Targeted Radionuclide Delivery

**DOI:** 10.1155/2011/368535

**Published:** 2011-01-17

**Authors:** Eliot. P. Botosoa, Mike Maillasson, Marie Mougin-Degraef, Patricia Remaud-Le Saëc, Jean-François Gestin, Yannick Jacques, Jacques Barbet, Alain Faivre-Chauvet

**Affiliations:** ^1^Centre de Recherche en Cancérologie Nantes-Angers (CRCNA), Université de Nantes, Inserm, UMR 892, Institut de Recherche Thérapeutique de l'Université de Nantes, 8 quai Moncousu, BP 70721, 44007 Nantes Cedex 1, France; ^2^Plateforme Interactome & Puces à Protéines Biogenouest, Institut de Recherche Thérapeutique de l'Université de Nantes, 8 quai Moncousu, BP 70721, 44007 Nantes Cedex 1, France

## Abstract

Targeted PEGylated liposomes could increase the amount of drugs or radionuclides delivered to tumor cells. They show favorable stability and pharmacokinetics, but steric hindrance of the PEG chains can block the binding of the targeting moiety. Here, specific interactions between an antihapten antibody (clone 734, specific for the DTPA-indium complex) and DTPA-indium-tagged liposomes were characterized by surface plasmon resonance (SPR). Non-PEGylated liposomes fused on CM5 chips whereas PEGylated liposomes did not. By contrast, both PEGylated and non-PEGylated liposomes attached to L1 chips without fusion. SPR binding kinetics showed that, in the absence of PEG, the antibody binds the hapten at the surface of lipid bilayers with the affinity of the soluble hapten. The incorporation of PEGylated lipids hinders antibody binding to extents depending on PEGylated lipid fraction and PEG molecular weight. SPR on immobilized liposomes thus appears as a useful technique to optimize formulations of liposomes for targeted therapy.

## 1. Introduction

The development of liposomes capable of targeting cells has been an objective since the 80s [1, 2]. The most prevalent method is to conjugate antibodies or antibody-based constructs (e.g., fragments or single chain Fv) directly on their surface (i.e., immunoliposomes). However, the ability of immunotargeted liposomes to deliver high doses of drugs or radioactivity to tumor cells *in vivo* remains to be demonstrated, partly because it is difficult to include all necessary features, that is, long circulation times, stable drug encapsulation or radiolabeling with high activities, and efficient antibody targeting in the liposomes preparation [3]. 

Other antibody constructs, such as bispecific antibodies, provide an alternative way to specifically target liposomes to cancer cells [4]. The bispecific antibody is used here as a pretargeting agent. It recognizes both a tumor-specific antigen and a small molecule (the hapten) used as a tag to the liposome membrane. The pretargeting system presents the advantage of using a stable bispecific antibody and liposomes that can be loaded extemporaneously with drugs or radionuclides, whereas stability and loading of immunoliposomes may be a problem. We have developed a liposomes radiolabeling method which is based on an active-loading approach for obtaining high specific activity-labeled liposomes [5]. Thus, the use of liposome as delivery systems represents an attractive alternative to vehicle important quantities of radionuclides. 

Recent formulations of liposomes prevent their opsonization by serum proteins and thus enhance residence time in the bloodstream. This is obtained by the addition of PEG functionalized lipids in their composition [6–8]. Different PEGylated liposomes formulations bearing the DTPA-indium hapten at their surface have been tested. Such PEGylated liposomes, also referred to as stealth liposomes, containing doxorubicin and a few other drugs have been approved for marketing. Liposomes containing 1.5%, 5%, or 8% PE-PEG were analysed for blood clearance over 24 h after injection in mice. Rapid elimination of conventional liposomes and 1.5% PEGylated liposomes was observed. Incorporation of 5% PEG in liposome considerably increased the retention time in bloodstream. The experiment showed identical half life and clearance (13,06 h and 0.16 mL/h or 13,89 h and 0.20 mL/h, resp.) for 5 and 8% DSPE-PEG, indicating that 5% DSPE-PEG is sufficient to obtain a maximum blood residence time [9]. Nevertheless, preliminary *in vivo* results have shown an improvement by only a factor of 1.7 between passive tumor targeting (absence of bispecific antibody) and active targeting of the liposomes by prior injection of a bispecific antibody binding carcinomembryonic antigen (CEA) on one arm and the DTPA-indium hapten on the other, in a model of CEA-positive tumor xenografts in the mouse. Passive targeting of the liposomes through the well-known enhancement permeability and retention effect [5] is very significant, and, therefore, to be interesting, active targeting of the liposomes to the tumor sites must be more efficient than what we observed with these hapten-tagged PEGylated liposomes. It is long known that PEGylation can hinder specific recognition between immunoliposomes and target cells [10]. Steric hindrance may also be the reason for the poor enhancement of tumor uptake caused by the bispecific antibody. Since this phenomenon has never been studied in a quantitative manner, we decided to use surface plasmon resonance (SPR) to characterize the specific interactions between the antihapten antibody and hapten-tagged liposome as a model of specific immunologic interaction at the liposome surface in the presence of varying amounts of PEGylated lipids and various PEG chain lengths. SPR is a technique that is frequently applied for measuring binding rate constants between two interacting entities, generally proteins. Its most obvious advantages over other techniques are: direct and rapid determination of association and dissociation rates of binding process and no need of labeling liposomes. Several studies have demonstrated that the technique is sensitive enough to monitor interactions between solutes and lipid bilayers like liposomes. Artificial bilayer lipid membranes (BLMs) have been extensively used to mimic biological cell membranes for studying membrane processes such as signal transduction, ligand-receptor interactions, and ion transport through cell membranes [11–13]. Recent advances in the preparation of stable membrane-like surfaces and the commercialization of sensor chips has enabled widespread use of SPR in analyzing these protein-membrane interactions in an environment that closely resembles our *in vivo* situation [14–16]. In this study, tethered bilayer membrane on CM5 chips and nonfused liposomes immobilized on L1 chips have been used to monitor by SPR the binding of antibodies to conventional and PEGylated DTPA-indium-tagged liposomes. We compared several liposomes formulations composed of distearoylphosphatidylcholine (DSPC), cholesterol (Chol), DSPE-DTPA that varied in their PEG content and molecular weight (2000, 1000, or 750). Binding kinetics of a specific anti-indium-DTPA antibody (clone 734) were monitored using the BIAcore system and the kinetic parameters were calculated by curve fitting.

## 2. Materials and Methods

### 2.1. Materials and Equipment

The purified MAb 734 IgG, with binding specific for the DTPA-indium complex, was kindly provided by IBC Pharmaceuticals (Morris Plains, NJ).

All chemicals were dissolved in sterile water (versol or versylene, FRESENIUS, France). Phosphate buffered saline (PBS 9.55 g·L^−1^, PBS DULBECCO) was supplied by BIOCHROM AG, (Berlin, Germany). 0.4 M N-ethyl-N-(3-dimethylaminopropyl)-carbodiimide hydrochloride and 0.1 M N-hydroxysuccinimide (NHS) were obtained from GE Healthcare. Dimyristoyl-L-*α*-phosphatidylethanolamine (DMPE), Triton-X100 (t-octylphenoxypolyethoxyethanol) and stable indium-115 chloride (^115^In) were purchased, respectively, from Sigma-Aldrich and Sigma Ultra. Radioactive indium-111 chloride (^111^In) was purchased from Mallinckrodt (Petten, The Netherlands). 

Other phospholipids used to prepare liposomes were: 1,2-Distearoyl-sn-glycerol-3-phophoethanolamine-N-[Methoxy(Polyethylene glycol)-2000] M.W : 2805.54 (DSPE-PEG2000), 1,2-Distearoyl-sn-glycerol–3-phophoethanolamine-N-[Methoxy(Polyethylene glycol)-1000] M.W : 1631.37 (DSPE-PEG1000), 1,2-Distearoyl-sn-glycerol-3-phophoethanolamine-N-[Methoxy(Polyethylene glycol)-750] M.W : 1528 (DSPE-PEG750), and 1,2-Distearoyl-sn-glycerol-3-phophoethanolamine-N-[Methoxy(Polyethylene glycol)-550] M.W : 1351.78 (DSPE-PEG550) were purchased from Avanti Polar Lipids (Alabaster, AL, USA). (DSPE-DTPA) was synthesized by Ecole Nationale Supérieure de Chimie de Rennes (France). Vesicle extruder and filter supports were purchased from Avanti Polar Lipids, Inc. Polycarbonate membranes for vesicle extrusion (100 nm or 200 nm pore size, Nucleopore) were from Whatman. All phospholipids were dissolved in 9 : 1 chloroform/methanol mixture (HPLC grade, Carlo Erba and Fisher Scientific, resp.).

### 2.2. MAb 734 Equilibrium Binding Assays in Coated Tubes

Avidin-coated tubes saturated with bovine serum albumin (BSA) were used for equilibrium affinity constant determinations of the anti-DTPA-indium antibody (MAb 734) in competition experiments between DTPA-^111^In as a tracer and stable DTPA-metal complexes. Briefly, 1 mL of a 50 ng/mL solution of biotinylated Mab 734 Fab fragment was incubated overnight at 4°C in the avidin coated tubes. Just before use, the tubes were washed with NaCl 0.9%-Tween 20 0.05%. DTPA (0.1 nmol) was labeled with commercial indium-111 chloride (5 × 10^7^ cpm) and used as a tracer (15000 cpm in a total incubation volume of 0.3 mL). Incubation with varying concentrations of stable DTPA-metal or EDTA-metal competitors was performed overnight at 4°C in PBS supplemented with BSA. Tubes were then counted after two rapid washes with 2 mL of NaCl-Tween.

### 2.3. DMPE Solubilization for CM5 Coating

Dimyristoyl-L-*α*-phosphatidylethanolamine (DMPE) was thoroughly mixed with PBS containing 1% Triton-X100 (t-octylphenoxypoly-ethoxyethanol) to a final concentration of 1 mg·mL^−1^, followed by at least three freeze-thaw cycles, ultrasonication, and incubation at 55°C.

### 2.4. Liposomes Preparation and Characterization

For vesicles preparation, the desired phospholipids (DSPC) in organic solvent CHCl_3_/MeOH (9 : 1) were transferred to a 10 mL round bottom flask and the solvent was evaporated to dryness. PBS was then added to the lipid film for a final lipid concentration of 20 *μ*mol·mL^−1^.

Large unilamellar vesicles (LUVs) composed of DSPC, DSPC/DSPE-DTPA (98 : 2 molar ratio) or DSPC/Chol/DSPE-DTPA (68 : 30.5 : 1.5 molar ratio) were prepared according to the lipid film hydration method [17] followed by extrusion. Typically, for the nonfused liposomes, 13.5 *μ*mol of phospholipids, 6.6 *μ*mol of cholesterol, and 0.3 *μ*mol of phospholipids coupled to the chelating agent (DTPA) were dissolved in chloroform/methanol (9 : 1 v/v) in a 10 mL round bottom flask. DSPE-PEG2000 (0.5 mol%, 1.5 mol%, 2.5 mol%, 3.5 mol%, or 5 mol%) were included in the preparation according to the necessity of experimentation.

A thin dry film of lipids was obtained by evaporation of the solvents in a rotary evaporator. Hydration of the dry lipids was accomplished by addition of 1 mL of aqueous phase and maintained above the gel crystal transition temperature of the lipids during all the hydration procedure. To this effect, the flask containing the liposomes suspension was mixed during 2 h on a rotary evaporation system without vacuum, at room temperature for conventional liposomes (DSPC), and 74°C for DSPC/Chol/DSPE-DTPA PEGylated liposomes. Typically, the final concentration of the liposomes suspension was 20 *μ*mol of lipids per mL of aqueous phase.

To obtain small and homogeneous vesicles, the liposomes suspension was sonicated times to time in a bath-type sonicator then 20 times extruded through Nucleopore 100 nm polycarbonate filters using a manual thermostat heated extrusion device at room temperature for conventional liposomes and at 74°C for PEGylated liposomes [18]. The size and polydispersity of the vesicles were measured by dynamic laser light-scattering system using a Malvern High Performance Particle Sizer (HPPS-ET, Instrument SA, UK). Measurements were performed in triplicate after dilution of the suspension in water. The mean size were 101 ± 4 nm (polydispersity index <0.1) for conventional liposomes and 107 ± 3 nm (polydispersity index <0.1) for PEGylated liposomes with all concentrations of PEG2000.

### 2.5. 115 In Loading Procedure

DTPA functionalized liposomes were prepared in citrate (0.10 M)/acetate (0.15 M) buffer, pH = 5.3. Nonradioactive indium (^115^In) chloride in HCl 0.02 N was added with a ratio of 10 indium molar equivalents per mole of lipids, and the mixture was incubated for 2 hours at 37°C. Then, ^115^In-loaded liposomes were separated from free indium by gel filtration chromatography using a PD-10 column eluted in PBS.

### 2.6. Formation of Lipid Planar Bilayers on CM5 Chips

Freely accessible terminal carboxyl groups of the dextran layer were activated with N-ethyl-N'-(3-dimethylaminopropyl)-carbodiimide hydrochloride) (EDC) and N-hydroxysuccinimide (NHS). The primary amine of dimyristoyl-L-*α*-phosphatidylethanolamine (DMPE) was then reacted with the activated succinimide esters overnight at 55°C. This reaction yields the proximal monolayer of the lipid membrane that is covalently attached to the dextran layer on the gold surface. Then, the DMPE-coated surface was thoroughly rinsed with distilled water and the chip was docked again in the BIAcore instrument. All flow cells were washed three times with 30 *μ*L 100 mM NaOH (flow rate 30 *μ*L/min). DMPE coupling provides the highly hydrophobic surface necessary for the subsequent functionalization of the tethered membrane by spontaneous vesicle spreading. Lipid vesicles or liposomes were then spread on the DMPE layer to constitute the bilayer. Briefly, liposomes (1 mg/mL in PBS) were injected over the hydrophobic surface for four to ten minutes at a flow rate of 10 *μ*L/min.

### 2.7. Binding of Intact Liposomes to L1 Chips

The BIAcore 3000 instrument equipped with the L1 chip was used for Surface Plasmon Resonance measurement of antibody binding to nonfused liposomes. The surface of the chip was conditioned with three consecutive injections for 1 min at 30 *μ*L/min of isopropanol/50 mM NaOH (2/3, v/v). 

Liposomes (1 mg/mL in PBS) were deposited on three flow cells for 10 min at flow rate of 5 *μ*L/min. The liposomes surface was washed with NaOH 100 mM for 1 min at 30 *μ*L/min. Bound liposomes could be removed from the L1 surface at the end of the experiments by two 1-minute injections of 50 mM NaOH : isopropanol (2/3, v/v) followed by two injections of Chaps 2% (w/v). Surface binding of L1 biosensor chip can be regenerated as often as needed.

### 2.8. Atomic Force Microscopy (AFM)

A multitask AFM CP was used for AFM imaging in the tapping mode and topographic measurements. Typically for the analysis, we observed the presence of a tethered lipid bilayer modified area and a nonmodified surface.

### 2.9. Kinetic Measurements

For all measurements, the flow rate was fixed at 60 *μ*L/min. Serial two-fold dilutions of MAb 734 were prepared (750 nM to 0.78 nM) and injected over on either the tethered planar bilayer on CM5 sensor chip or nonfused liposomes immobilized on the L1 sensor chip. Dilutions of MAb 734 were injected from low to high concentration in a single-cycle kinetic (SCK) mode with association phases monitored for 3 min at 60 *μ*L/min and allowing 4 min dissociation phases.

### 2.10. Data Analysis

The resulting sensorgrams were fitted using a mathematical program based on single cycle kinetic model implemented in BIAeval 4.1 software (BIAcore).

## 3. Results

### 3.1. MAb 734—DTPA-Indium Binding Characterization

**Figure 1:**
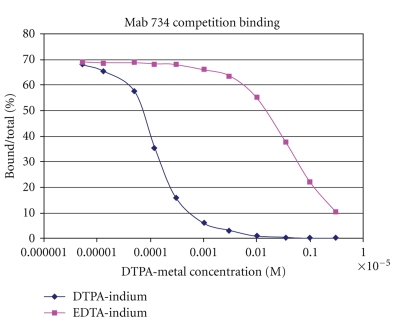
MAb 734 binding with DTPA-indium hapten. Inhibition of DTPA-^111^Indium binding to biotinylated MAb734 coated to avidin tubes as a function of DTPA-indium or EDTA-indium concentration. The equilibrium affinity constants were calculated from the data using standard Scatchard analysis.

The MAb 734 was originally screened for its binding to soluble DTPA-indium complex [19]. Competition experiments (Figure 1) using tubes coated with MAb 734 allowed the equilibrium dissociation constant to be determined as 0.3 nM at 4°C.

### 3.2. CM5 Bilayer

#### 3.2.1. Formation of Lipid Planar Bilayers on CM5 Sensor Chip

**Figure 2:**
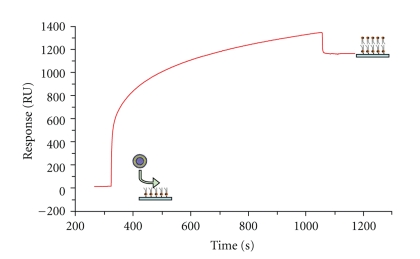
Tethered planar bilayer formation on DMPE functionalized CM5 chip. A BIAcore CM5 chip was coated with DMPE as described in materials and methods. The functionalized chip was treated with DSPC/DSPE-DTPA containing vesicles at a 5 *μ*L/min flow rate. The sensorgram shows the spreading of liposomes to a level of 1100 RU and the formation of a stable planar bilayer.

DMPE was used for the setup of tethered artificial membranes by chemical coupling of the primary amino groups with succinimide esters of the dextran carboxylate groups. Then, the three formulations of liposomes, PEGylated, and conventional were spread on the DMPE monolayer after rinsing with PBS.  Figure 2 shows that liposomes give a stable signal of 1100 RU. This value is in agreement with the expected RU of the second monolayer coating of the CM5 sensor chip functionalized with DMPE.

**Figure 3:**
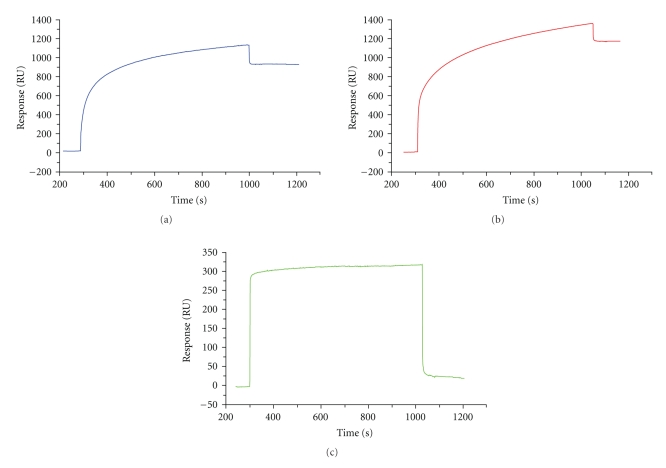
Formation of tethered planar bilayers on DMPE-functionalized CM5 chip with liposomes of different lipid compositions. A DMPE-coated chip was treated with liposomes prepared with 4 different lipid compositions: (a) DSPC-containing lipid vesicles that spread and formed a 900 RU tethered planar bilayer, (b) DSPC/DSPE-DTPA-indium-containing lipid vesicles that spread and formed a 1100 RU tethered planar bilayer, and (c) DSPC/DSPE-DTPA-indium/DSPE-PEG-containing lipid vesicles that were unable to spread on the hydrophobic DMPE monolayer.

Figure 3 emphasizes that (a) DSPC-containing lipid vesicles spread and formed a 900 RU tethered planar bilayerand (b) DSPC/DSPE-DTPA-Indium-containing lipid vesicles also spread but formed a 1100 RU tethered planar bilayer whereas (c) DSPC/DSPE-DTPA-Indium/DSPE-PEG2000-containing lipid vesicles were not able to spread on the DMPE-monolayer.

Our formulation (DSPC/DSPE-DTPA-Indium-containing lipid vesicles) induced a gain of 200 RU compared to the standard formulation (DSPC-containing lipid vesicles) (Figures 3(a) and 3(b)). 

Although conventional liposomes (non-PEGylated) gave satisfactory results, it was not possible to create a lipid planar bilayer with the PEGylated liposomes (Figure 3(c)). This can be explained by the fact that PEG-chains constitute a barrier against spreading on the DMPE layer. 

A CM5 sensor chip coated with the model bilayer obtained by fusing conventional liposomes to the DMPE layer was examined with an Atomic Force Microscope (AFM). Images clearly showed the topographical structure of lipid planar bilayers overlaying the dextran matrix and no liposomes stuck to the dextran as single particles confirming previous findings on either the characterization of planar supported bilayers [20] or the behavior adopted by liposomes adsorbed on CM5 and L1 sensor chips with modified dextran matrix [15, 21]. We could assume that liposomes fuse to form a lipid planar bilayer on the top of the dextran matrix which is the upper component of CM5 chips. Moreover, it has already been demonstrated that liposomes adsorbed on L1 chips surfaces may remain as intact single vesicles.

#### 3.2.2. MAb 734 Binding to DTPA-Indium Coupled to Lipid Planar Bilayer

The affinity of MAb 734 to the DTPA-indium functionalized lipid planar bilayer was tested on a BIAcore 3000 instrument in a single cycle kinetic model. The antibody bound the DTPA-indium hapten coupled to the DSPE layer without binding to the control DSPC layer. The binding was followed in real-time by a sensorgram resulting from the single-cycle kinetics assays on the functionalized bilayer after subtraction of the control DSPC flow cell. The interaction is highly specific in this range of concentration. The association and dissociation rate constants were calculated using the single cycle kinetics model also called “titration kinetics model.” The kinetic constants for MAb 734 binding to the DTPA-indium-functionalized bilayer calculated using this procedure of global fitting are listed in Table 1. The ratio of the kinetic constants (*k*_off_/*k*_on_) provided a *K*_*D*_ value, 1.6 nM, similar to those determined in the equilibrium binding experiments.

### 3.3. L1 Chip

**Figure 4:**
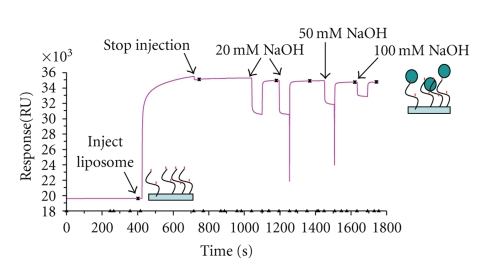
Deposition and absorption of liposomes on an L1 chip. Liposomes were deposited on the L1 chip for 5 min at 5 *μ*L/min in PBS. The concentration of lipids was 1 mg/mL. The flow rate was changed to 30 *μ*L/min after the deposition, and liposomes were washed with three consecutive injections of NaOH in increasing concentration (20, 50, and 100 mM). All vesicles were in PBS during the injection. A control surface was prepared by loading PEGylated liposomes without DTPA-indium on the first flow cell of a L1 sensor chip (DSPC/Chol/DSPE-PEG2000).

A control surface was prepared by loading vesicles without DTPA-Indium on the first flow cell of a L1 sensor chip, resulting in the deposition of 15000 RU. As indicated in Figure 4, conventional and PEGylated liposomes were properly adsorbed on the sensor chip with approximately the same level of absorbance (15–16000 RU). It was not possible to rule out vesicle fusion on the surface of the L1 chip by a direct observation with the AFM. We assume that the liposomes are adsorbed intact. In addition, previous published data “strongly” suggested that vesicles remain intact once bound to the lipophilic anchors on the surface of L1 chips [22, 23]. Therefore, these vesicles still maintain their biophysical properties. This finding has been confirmed by the characterization of calcein-loaded immobilized liposomes [24]. 

Then, MAb 734 binding kinetics to the surface of L1 chip which has been coated with different types of liposomes (DSPC/Chol/DSPE-DTPA (68 : 30.5 : 1.5), DSPC/Chol/DSPE-DTPA/DSPE-PEG2000 (63 : 30.5 : 1.5 : 5)) was monitored as above.

The calculated affinity constant (*K*_*D*_ = 1.6 × 10^−9^ M) for conventional liposomes was exactly the same as the value obtained with planar bilayer formed by conventional liposomes on CM5 (Table 1). The surface of the first flow cell coated with vesicles without DTPA-indium was used as a nonspecific binding control. In addition, antibody binding was observed only on liposomes surfaces functionalized with DTPA loaded with indium (data not shown).

#### 3.3.1. Influence of DSPE-PEG Molar Fraction

**Figure 5:**
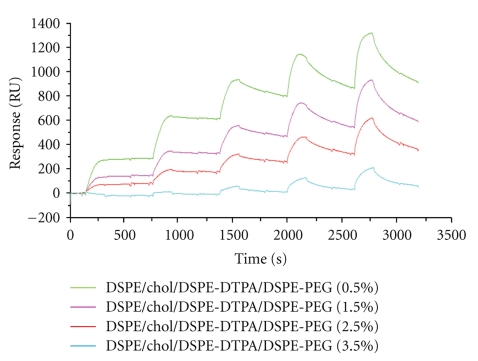
MAb 734 binding to DTPA-indium-tagged liposomes adsorbed onto a L1 chip. Kinetic titration series of MAb 734 on DSPC/DSPE-DTPA-indium-containing liposomes with various DSPE-PEG 2000 concentrations deposited on an L1 chip. The liposomes were deposited on the L1 Chip at the same level of Resonance Units, approximately 15 000 RU. For each formulation, MAb 734 was prepared in two-fold serial concentration series (720 nM to 2.185 nM) and was injected at a 60 *μ*L/min flow rate across the liposomes adsorbed on the L1 chip surface for 3 min. At the end of each injection, the dissociation phase was set to 4 min.

The binding responses shown in Figure 5 illustrate that DTPA-indium-functionalized liposomes prepared with DSPE-PEG2000 at various concentrations (0.5%–1.5%–2.5%–.5%) bound the antibody with an affinity that strongly decreased with the DSPE-PEG2000 fraction (MAb 734 antibody-binding responses were normalized for the level of liposomes captured on each surface, making it possible to compare the binding results directly). This effect was observed when the DTPA-indium hapten was directly coupled to DSPE (DSPE-DTPA). Using the same fitting procedure (SCK model), the kinetic constants for MAb734 binding were calculated for the different percentages of PEGylated lipid in the liposomes preparations (Table 2). Thus, the higher affinity was observed for the non-PEGylated liposomes. When the concentration of DSPE-PEG2000 was increased from 0 to 3.5%, *k*_on_ reduced about 40 times while *k*_off_ increased about 3 times. The Rmax value seems to follow the same decrease as the association rate. From these findings, we can assume that the DTPA-indium haptens are masked by the PEGylated chains of DSPE-PEG2000. More precisely, the diminution of association rate constant and Rmax can be almost totally explained by the steric effects of the PEGylated chains that reduce the diffusion factor. The faster dissociation rates may be attributed to a decrease of rebinding during the dissociation phase that can also be explained by steric hindrance. Both effects combined in increasing the dissociation constant from 1.6 nM in the absence of DSPE-PEG to over 1 *μ*M with 3.5% of DSPE-PEG2000.

#### 3.3.2. Influence of DSPE-PEG Chain Size

The binding responses shown in Table 3 emphasize that DTPA-indium-functionalized liposomes prepared with DSPE-PEG at various sizes (DSPE-PEG750–DSPE-PEG1000) bound the antibody with a much higher affinity for DSPE-PEG750 compared to DSPE-PEG2000 at the same concentration (2.5% of DSPE-PEG). The antibody was also able to bind the liposomes with a higher concentration of DSPE-PEG750 (6% and 8% of DSPE-PEG). Liposomes PEGylated with PEG1000 gave an affinity which was intermediate but higher than the one obtained with DSPE-PEG2000.

## 4. Discussion

The fundamental properties of unconjugated liposomes (e.g., size, surface charge, PEGylation, and membrane fluidity) that largely determine their fate *in vivo* have been identified [10, 25], and their effect on liposomes biodistributions and pharmacokinetics has been studied and understood to a great extent. However, the presence of surface conjugated-ligands (antibodies, protein fragments, and haptens) in targeted liposomes introduces additional complexities in their interactions with the biological milieu. A better understanding of these interactions will result in better targeted liposomes for maximum targeting specificity. At this point, it is clear that addition of PEG to the liposomes surface is needed to prevent opsonisation, fast uptake of the liposomes in the liver, and rapid clearance. Introducing immunospecific ligands in the liposome membrane can target the liposomes and their content, but PEG chains interfere with antibody recognition. This steric hindrance is observed when an antibody is attached to the liposomes surface [10] but also, as shown in this paper, when the liposome is tagged with a small molecular weight ligand to be recognized by an antibody or, as described by Cao and Suresh [4], by a bispecific antibody. This study also demonstrates that biosensors and SPR may be used to quantify this phenomenon of steric hindrance as a function of the fraction of PEGylated lipids added to the liposomes and as a function of the length of the PEG chains. 

Using CM5 chips, tethered bilayers are obtained upon addition of non-PEGylated liposomes. CM5 chip provides us with the development of a tethered bilayer obtained by the spreading of non-PEGylated liposomes that yielded us to determine information on kinetics and thermodynamics. This model offered the advantages of controlling more precisely the bilayer formation and above all the quantity and the saturation degree of hapten used for the calculation of binding rates. The antihapten antibody then binds to the hapten coupled to phospholipids and incorporated into the liposomes preparation. The binding kinetics between MAb 734 and the indium-DTPA hapten bound to these non-PEGylated phospholipid bilayers showed specific binding with an affinity value of 1.6 × 10^−9^ M, close to that measured in a completely different system of immobilized antibody and soluble radiolabeled indium-DTPA hapten, which may be considered as a mirror situation. This first experiment provides us with reference-binding rates and dissociation constants obtained both by kinetics and equilibrium measurements. Unfortunately, DPSE-PEG containing liposomes appeared not to bind and fuse on the surface of these CM5 chips. This may be easily explained by the hydrophilic barrier created by PEG chains at the surface of the liposomes preventing the interaction with the hydrophobic surface of the chip. Thus, L1 chips were used and shown capable of binding both conventional and PEGylated liposomes, independently of the DSPE-PEG2000 molar ratio, PEG chain length, and the addition of hapten-bearing phospholipids. Although AFM could not demonstrate directly that the liposomes attached to the L1 chips remain intact, indirect evidence has been published in the literature in favor of this hypothesis [22–24]. L1 chips were coated with liposomes prepared with different lipid compositions to very similar RU signals. It is difficult to ascertain that this means that the number of liposomes or the number of indium-DTPA molecules attached to the chip is identical with PEGylated and non-PEGylated liposomes. However, this means that the orders of magnitude of these numbers are similar. Besides, the kinetic analysis used to derive binding constants is not dependent on this number. These differences in binding affinities must reflect steric hindrance from the PEG chains and not a problem of liposomes capture. Thus binding of preformed liposomes of various compositions to L1 chips provides a robust and versatile system for *in vitro* binding studies of antibodies directed against liposome-bound antigens, using SPR.

The incorporation of PEGylated lipids in the liposome membrane hinders antibody binding in a PEGylated lipid fraction-dependent manner. The concentration and the chain length of PEGylated lipids are limiting factors for antibody-liposome interaction. Results from these binding studies show that hapten-tagged vesicles prepared with DSPE-PEG2000 at a ratio equal to or greater than 5% are poorly recognized by the antihapten antibody. The relative affinity of the antibody for hapten-bearing liposomes decreased from 1.6 × 10^−9^ M to 1.1 × 10^−7^ M when the PEGylated lipid percentage increased from 0% to 5%. We have looked into the variations of speed and diffusion coefficients in real-time. Rate constants measured by SPR revealed decreased diffusion coefficients of the antibody with vesicles containing various concentrations of DSPE-PEG2000 that translated in *k*_on_ values decreasing with the PEG concentration. Similarly, Rmax value showed a decrease of the availability of haptens.

All these phenomena could be physically explained by the relative pliability and flexibility of PEG chain and its unspecific interactions in equilibrium with the positive charge molecules present at the surface of liposomes. PEG chain with a relatively long size induces a steric hindrance, a diffusing difficulty of the antibodies to the hapten site. The masking of these haptens results in *k*_on_ decreasing value and a decrease of Rmax. Nevertheless, once the antibodies are bound to their haptens the intensity of the interactions is not modified and only the rebinding capacity is diminished, that is traduced by a less increasing of *k*_off_ value.

Liposomes prepared with shorter PEG chains—DSPE-PEG750 or DSPE-PEG1000—have more tightly bound the anti-hapten antibody, thus showing less steric hindrance. The shorter the chain, the easier the antibody MAb 734 diffuses, the higher the *k*_on_, and the higher the affinity for the antibody-hapten interaction. These three effects may be the result of two phenomena: PEG chain sweeping is influenced by the chain length and the formation of a tight mesh of PEG chains is influenced by the PEG concentration. Clearly, size and concentration of PEG chains limit the mass transfer of antibodies to their binding sites. Shorter PEG chains—even at high concentrations—should improve *in vivo* targeting because the affinity decreased only to 2.5 × 10^−8^ M and 5.5 × 10^−8^ M with, respectively, 8% of DSPE-PEG750 or 6% of DSPE-PEG1000.

As the shorter PEG chains are more rigid, they are less capable of sweeping and unspecifically interacting with the liposomes. The less PEG chain interfere with the diffusion of antibodies and the masking of haptens, the less kinetics constant are modified and the more PEG concentration could be increased without loss in affinity. 

The first *in vivo* pretargeting experiments, with PEGylated radiolabeled liposomes prepared with 5% of DSPE-PEG2000 and bispecific antibodies, showed encouraging but insufficient results with a tumor uptake increased by a factor of 1.7, compared to passive targeting of conventional liposomes. The results of this study by SPR reveal and quantify a large loss of hapten-antibody affinity with such a formulation. The pharmacokinetic parameters of the other formulations have been evaluated (data not shown) showing reasonably long circulation times, particularly with liposomes containing 6% or 8% of shorter PEG chains (750 or 1000). Since these liposomes formulations show reduced steric hindrance for the hapten—antibody interactions, they will be tested for pretargeted delivery of radionuclides to tumors *in vivo*.

## 5. Conclusion

SPR biosensors, such as BIAcore, are most often used to measure the binding kinetics and affinity constants of molecular interactions. We describe here an application that could have a significant impact on the study of antibody/liposome interactions. Earlier studies demonstrated the feasibility of biosensor simulation for acquiring binding data and predicting targeting performance. The study of PEGylated liposome formulations with variable PEG 2000 fractions and different PEG chain sizes (PEG 1000 and PEG 750) by SPR prompted us to perform further pharmacokinetics experiments to obtain the necessary information to improve immunotargeting *in vivo*. This method will also be applied to other kinds of particulate nanovectors.

## Figures and Tables

**Table 1:** Analysis of MAb 734 binding on tethered DSPC/DSPE-DTPA-indium bilayer created on CM5 chip and of MAb 734 binding to DTPA-indium-tagged liposomes adsorbed onto a L1 chip via *k*_on_ (1/Ms), *k*_off_ (1/s), Rmax (RU), and *K*_*D*_ (M) parameters. MAb 734 was prepared in two-fold serial concentration series (720 nM to 2.185 nM) and was injected at a flow rate 60 *μ*L/min across the liposomes adsorbed L1 chip surface for 3 min. At the end of each injection, the dissociation phase was set to 4 min. Liposomes binding assay was only performed once with CM5 and duplicated with L1 chip. *k*_on_ and *k*_off_ values were obtained by nonlinear regression of experimental data fitted with the SCK mathematical model. The score of *χ* ^2^ (Chi2) is <5; it means that the model used adequately describes our data. *k*_on_, *k*_off_, and *K*_*D*_ values are the average ± standard deviations.

Chip	**k**_on_ (1/Ms)	**k**_off_ (1/s)	Rmax	**K**_**D**_ (M)
CM5	5.57 10^5^	8.99 10^−4^	80.7	1.60 10^−9^
L1	5.08 ± 0.04 10^5^	8.03 ± 0.03 10^−4^	1970	1.58 ± 0.01 10^−9^

**Table 2:** Analysis of MAb 734 binding with DSPC/Chol/DSPE-DTPA-indium/DSPE-PEG2000 liposomes via *k*_on_ (1/Ms), *k*_off_ (1/s), and *K*_*D*_ (M). The amount of DSPE-PEG contained in PEGylated liposomes varied from 0% to 5%. Liposomes were immobilized on L1 chip. The length of PEG chain was fixed unchanged at 2000. The DTPA amount was fixed. The score of *χ* ^2^ (Chi2) is <5; it means that the model used adequately describes our data. All experiments were duplicated. *k*_on_, *k*_off_, and *K*_*D*_ values are the average ± standard deviations.

Formulations (%DSPE-PEG2000)	*k*_on_ (1/Ms)	*k*_off_ (1/s)	*K*_*D*_ (M)
0%	5.08 ± 0.04 10^5^	8.03 ± 0.03 10^−4^	1.59 ± 0.01 10^−9^
0.5%	3.31 ± 0.38 10^5^	8.03 ± 0.07 10^−4^	2.45 ± 0.26 10^−9^
1.5%	1.52 ± 0.23 10^5^	1.21 ± 0.13 10^−3^	8.04 ± 0.39 10^−9^
2.5%	6.01 ± 0.07 10^4^	2.06 ± 0.05 10^−3^	3.28 ± 0.12 10^−8^
3.5%	1.02 ± 0.25 10^4^	2.68 ± 0.02 10^−3^	2.78 ± 0.63 10^−7^
5%	/	/	/

**Table 3:** Data of *K*_*D*_ (M) and Rmax (RU) resulted from MAb 734 binding with DSPC/Chol/DSPE-DTPA-indium/DSPE-PEG750 and DSPC/Chol/DSPE-DTPA-indium/DSPE-PEG1000 liposomes in order to emphasize the influence of PEG chain size. Three different concentrations of DSPE-PEG (2.5%, 6%, and 8%) were studied for each size of PEG chain. The score of *χ* ^2^ (Chi2) is <5; it means that the model used adequately describes our data. Experiments were duplicated. K_D_ value is the average ± standard deviations.

%DSPE-PEG	2.5%		6%		8%	
PEG Size	*K*_*D*_ (M)	Rmax (RU)	*K*_*D*_ (M)	Rmax (RU)	*K*_*D*_ (M)	Rmax (RU)
750	2.29 ± 0.11 10^−9^	1081	8.57 ± 0.08 10^−9^	902	2.51 ± 0.15 10^−8^	845
1000	4.25 ± 0.21 10^−9^	688	5.48 ± 0.22 10^−8^	529	1.15 ± 0.44 10^−7^	424

## Abbreviations Used

AFM: Atomic force microscopy
BLM: Bilayer lipid membrane
CM5: Carboxymethylated dextran matrix
DMPE: Dimyristoyl-L-*α*-phosphatidylethanolamine
DTPA: Diethylenetriaminepentaacetate
DTPA-In: Diethylenetriaminepentaacetate indium complex
DSPC: Distearyl-L-*α*-phosphatidylcholine
DSPE: Distearyl-L-*α*-phosphatidylethanolamine
Chol: Cholesterol
EDC: N-ethyl-N-(-3-dimethylaminopropyl)-carbodiimide hydrochloride
FRET: Fluorescence resonance energy transfer
*k*_off_: Dissociation rate
*k*_on_: Association rate
*K*_*D*_: Dissociation constant
LUV: Large unilamelar vesicle
MAb: Monoclonal antibody
NHS: N-hydroxysuccinimide
PBS: Phosphate buffered saline
PEG: Polyethylene glycol
PK: Pharmacokinetic
RU: Resonance unit
SPR: Surface plasmon resonance
SCK: Single cycle kinetic
Resp. Diff.: Response differential.
